# Molecular Level Characterisation of the Surface of Carbohydrate-Functionalised *Mesoporous silica* Nanoparticles (MSN) as a Potential Targeted Drug Delivery System via High Resolution Magic Angle Spinning (HR-MAS) NMR Spectroscopy

**DOI:** 10.3390/ijms23115906

**Published:** 2022-05-25

**Authors:** Karolina Krajewska, Anna M. Gołkowska, Maciej Nowak, Marta Kozakiewicz-Latała, Wojciech Pudło, Andrzej Żak, Bożena Karolewicz, Yaroslav Z. Khimyak, Karol P. Nartowski

**Affiliations:** 1 Department of Drug Form Technology, Wroclaw Medical University, Borowska 211A, 50-556 Wroclaw, Poland; karolina.krajewska@student.umw.edu.pl (K.K.); anna.golkowska@student.umw.edu.pl (A.M.G.); maciej.nowak@umed.wroc.pl (M.N.); marta.kozakiewicz-latala@umw.edu.pl (M.K.-L.); bozena.karolewicz@umed.wroc.pl (B.K.); 2 Department of Chemical Engineering and Process Design, Silesian University of Technology, M. Strzody 7 Str., 44-100 Gliwice, Poland; wojciech.pudlo@polsl.pl; 3 Faculty of Mechanical Engineering, Wroclaw University of Science and Technology (WUST), Wybrzeze Wyspianskiego 27, 50-370 Wroclaw, Poland; andrzej.zak@pwr.edu.pl; 4 School of Pharmacy, University of East Anglia, Norwich Research Park, Norwich NR4 7TJ, UK; y.khimyak@uea.ac.uk

**Keywords:** mesoporous silica nanoparticles, MSN, porous materials, functionalisation, NMR, HR-MAS NMR, surface

## Abstract

Atomistic level characterisation of external surface species of mesoporous silica nanoparticles (MSN) poses a significant analytical challenge due to the inherently low content of grafted ligands. This study proposes the use of HR-MAS NMR spectroscopy for a molecular level characterisation of the external surface of carbohydrate-functionalised nanoparticles. MSN differing in size (32 nm, 106 nm, 220 nm) were synthesised using the sol-gel method. The synthesised materials displayed narrow particle size distribution (based on DLS and TEM results) and a hexagonal arrangement of the pores with a diameter of ca. 3 nm as investigated with PXRD and N_2_ physisorption. The surface of the obtained nanoparticles was functionalised with galactose and lactose using reductive amination as confirmed by FTIR and NMR techniques. The functionalisation of the particles surface did not alter the pore architecture, structure or morphology of the materials as confirmed with TEM imaging. HR-MAS NMR spectroscopy was used for the first time to investigate the structure of the functionalised MSN suspended in D_2_O. Furthermore, lactose was successfully attached to the silica without breaking the glycosidic bond. The results demonstrate that HR-MAS NMR can provide detailed structural information on the organic functionalities attached at the external surface of MSN within short experimental times.

## 1. Introduction

Porous materials are solids that have pores, cavities, channels or fissures with depths greater than the width [1]. Three types of porous materials can be distinguished based on the pore size: microporous (pore diameter < 2 nm), mesoporous (pore diameter 2–50 nm) and macroporous (pore diameter > 50 nm) [1]. Mesoporous silica materials are synthesised using the sol-gel method in the presence of an acidic or basic catalyst [2,3,4]. The first synthesis of MCM−41 (Mobil Crystalline Materials/Mobil Composition of Matter)-type mesoporous silica particles was described in 1992 by Beck et al., which enabled particles characterised by a hexagonal pore arrangement with a pore size of 2–10 nm to be obtained [5,6]. In the MSN synthesis, the source of silica is usually TEOS (tetraethoxysilane), whose alkoxy groups undergo hydrolysis to silane groups, followed by homo- (reaction of silane groups with each other) and heterocondensation (reaction of alkoxy groups with silane groups) resulting in the formation of Si-O-Si siloxane bridges. Silica condensation occurs on a template (e.g., CTAB, cetyltrimethylammonium bromide) that forms cylindrically organised micelles. The surfactant (template) is removed by solvent extraction or calcination (at about 550 °C) leading to the final product. The particle size, pore size or shape can be modified by controlling the reaction conditions [7].

Mesoporous silica nanoparticles (MSN) have two surfaces, an outer surface and an inner surface, both covered with silanol (Si–OH) groups which allow their covalent conjugation with different types of functional groups such as carboxylates, amines, phosphonates, polyethylene glycol, octadecyl and carboxylate/octadecyl groups to name a few [8,9]. This property of MSN can be utilised for the synthesis of targeted drug delivery systems that can selectively bind to different cellular receptors or cell compartments [3,10]. One can distinguish between two methods of surface functionalisation: grafting and co-condensation. Co-condensation is a process that allows the direct functionalisation of silica during the material synthesis process. Typically, R–TMS or R–TES organosilanes, where R is the organic functional group and TMS or TES (trimethoxysilane and triethoxysilane, respectively) are added to the reaction either together with TEOS or immediately after the addition of TEOS, which ensures the incorporation of the organosilanes into the final product. This method is mainly preferred for the functionalisation of the inner surface of nanoparticles [11]. In contrast, grafting is a post-synthetic method of attaching organic or inorganic functional groups to the MSN surface. The reaction requires the condensation of organosilanes of the (R’O)_3_SiR type (where R stands for an organic radical) with reactive –Si–OH end groups in an anhydrous solvent, e.g., toluene. Grafting allows the preparation of hydrothermally stable MSN using classical synthesis methods and the subsequent selective functionalisation of the outer surface of these materials [7,12,13]. The grafting method was used in this work to functionalise nanoparticles with an amine group, galactose and lactose.

The unique properties of MSN, e.g., a high degree of ordering of their internal porous structure, hydrothermal stability and the possibility of surface modification has led to the application of MSN in many branches of science, including nanotechnology, medicine or pharmaceutical drug delivery [3,14,15,16,17]. A number of reports have demonstrated that the functionalisation of the MSN external surface is desirable for targeted cancer therapy applications as it increases the efficiency of material penetration into the tumour microenvironment [3,8,17,18,19,20,21]. MSN can also be used as novel carriers for nucleic acids in emerging gene therapies. Mukherjee and co-workers investigated the possibility of hepatitis C therapy using galactose-functionalised MSN with encapsulated shDNA. The presence of galactose at the silica surface was responsible for their selective uptake by liver cells that overexpress the asialoglycoprotein receptor. The plasmid shDNA encapsulated in the particles acted as a precursor for in situ siRNA production that binds to the target HCV mRNA sequence causing its degradation. Biodistribution studies in an in vivo model showed an increased uptake of galactose-modified silica in the liver compared to other organs. Further in vitro infection studies showed a significant (94%) reduction in viral RNA after shDNA delivery using Gal–AMSN in a HCV-JFH1 cell culture [21].

Understanding the spatial distribution of the functional groups at the silica surface is important for the rational design of new MSN for targeted drug delivery. The molecular structure of functionalised MSN can be analysed with solid-state NMR spectroscopy [22,23,24]. This method allows the chemical characterisation of the material structure along the surface species, enabling an investigation into the local environment of different nuclei (e.g., ^1^H, ^29^Si and ^13^C) to obtain distance information, the degree of organic functionalisation or the dynamics of functional groups [25]. The main limitation for the broad application of solid-state NMR spectroscopy for the characterisation of organically modified silica materials is the low sensitivity of the method which is mostly pronounced in the case of nanomaterials that possess functionalities exclusively at the external surface of the particles. Furthermore, the ^1^H NMR spectra of solids often show very broad lines due to the presence of strong dipolar couplings or orientation-dependent interactions (e.g., chemical shift anisotropy) even under RF decoupling or high rates of magic angle spinning (MAS). Structural analysis using the abovementioned methods is usually performed on dried powders, which does not reproduce the behaviour of MSN in an aqueous environment [26]. It should be considered that complex chemical transformations that do not occur in the solid phase may occur in biorelevant fluids (e.g., aggregation or opsonisation) [27]. For this reason, the development of novel methods for the structural analysis of functionalised nanomaterials is crucial for understanding their structure and dynamics in solutions. Recently, Tataurova et al. [28] authors used solution-state NMR spectroscopy to determine the number of bound and released ligands or groups that were exchanged upon the hydrothermal treatment of functionalised mesoporous silicas [29,30].

Advantages of solid- and solution-state NMR can be combined in the so called High Resolution Magic Angle Spinning (HR-MAS) technique that allows the analysis of materials that swell and become partially soluble or form true solutions even when some solids are still present. High resolution spectra are obtained due to spinning the sample at the ’magic angle’ (θ = 54.7°) with respect to the static magnetic field, which enables the strong anisotropic interactions to be overcome and as a consequence the width of the signals can be reduced. One of the advantages of this NMR technique is its timing. High resolution two-dimensional spectra (e.g., ^1^H-^13^C HSQC) in HR-MAS can be acquired in much less time (as short as 30 min) compared to the spectra obtained using solid state ^1^H-^13^C HETCOR NMR techniques (several hours to several days) [31,32].

**Figure 1:**
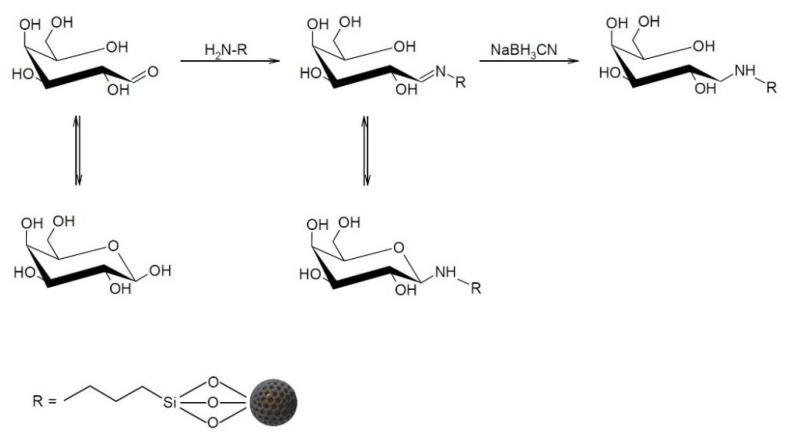
Reductive amination of galactose with amino-functionalised MSN.

**Scheme 1:**
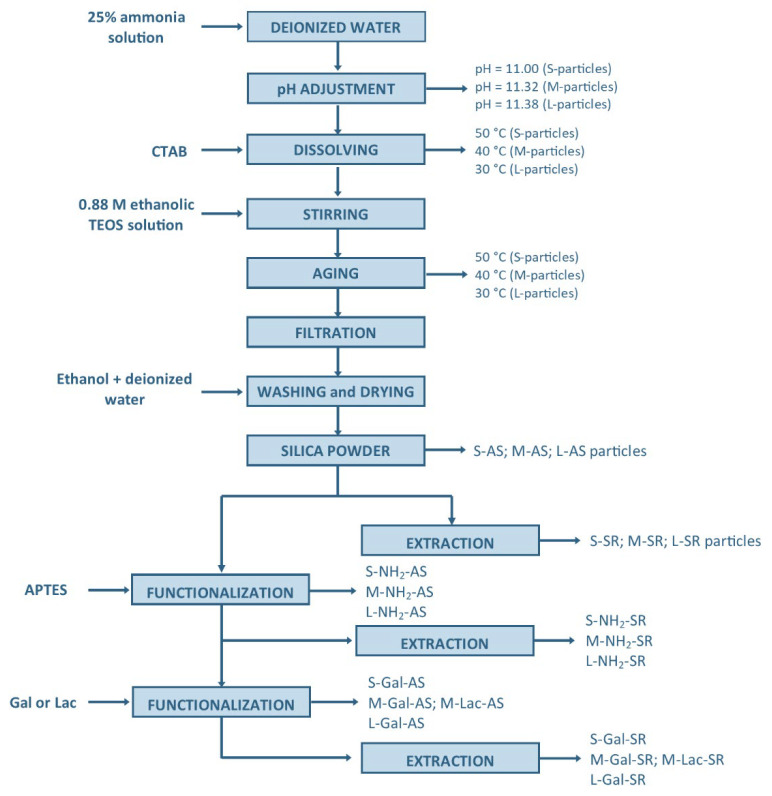
Flowchart presenting the synthesis, functionalisation and extraction steps required to obtain galactose and lactose-functionalised MSN particles. The abbreviations used for the materials labelling: S—small—32 nm particles; M—medium—106 nm particles; L—large—220 nm particles; AS—as synthesised; SR—surfactant removed; NH_2_—amino functionalised particles; Gal—galactose; Lac—lactose.

In this work we propose for the first time the application of HR-MAS NMR spectroscopy for the molecular level characterisation of the surface of functionalised MCM-41-type MSN in an aqueous medium in minutes. For this purpose, silica nanoparticles varying in size (32 nm, 106 nm, 220 nm) were synthesised and then their external surface was functionalised with APTES (amino group donor), galactose and lactose using the grafting method following the removal of the templating agent (Figure 1, Scheme 1). The size, shape and hexagonal structure of the particles were confirmed using DLS, low angle PXRD and N_2_ physisorption analysis corroborated with TEM imaging. The presence of organic functionalities at the silica surface was confirmed with ^1^H-^29^Si CP/MAS NMR, HR-MAS NMR spectroscopy and with FTIR. The development of a novel analytical technique for the molecular level understanding of functionalised nanomaterials enables a knowledge-based design of advanced functionalised nanocarriers for targeted drug delivery.

## 2. Results and Discussion

The successful synthesis of MSN (Scheme 1) was confirmed by a combination of analytical techniques which enabled an assessment of the size of the particles, BET surface area, pore volume and architecture as well as the formation of specific bonds. The main manuscript provides a detailed characterisation of the ‘M’ particles, while the analysis of ‘S’ and ‘L’ particles is provided in the Supplementary Materials (Figures S1–S10), which proves that the approach is applicable for nanoparticles with different sizes.

### 2.1. Particle Size, Morphology and Pore Architecture

**Figure 2:**
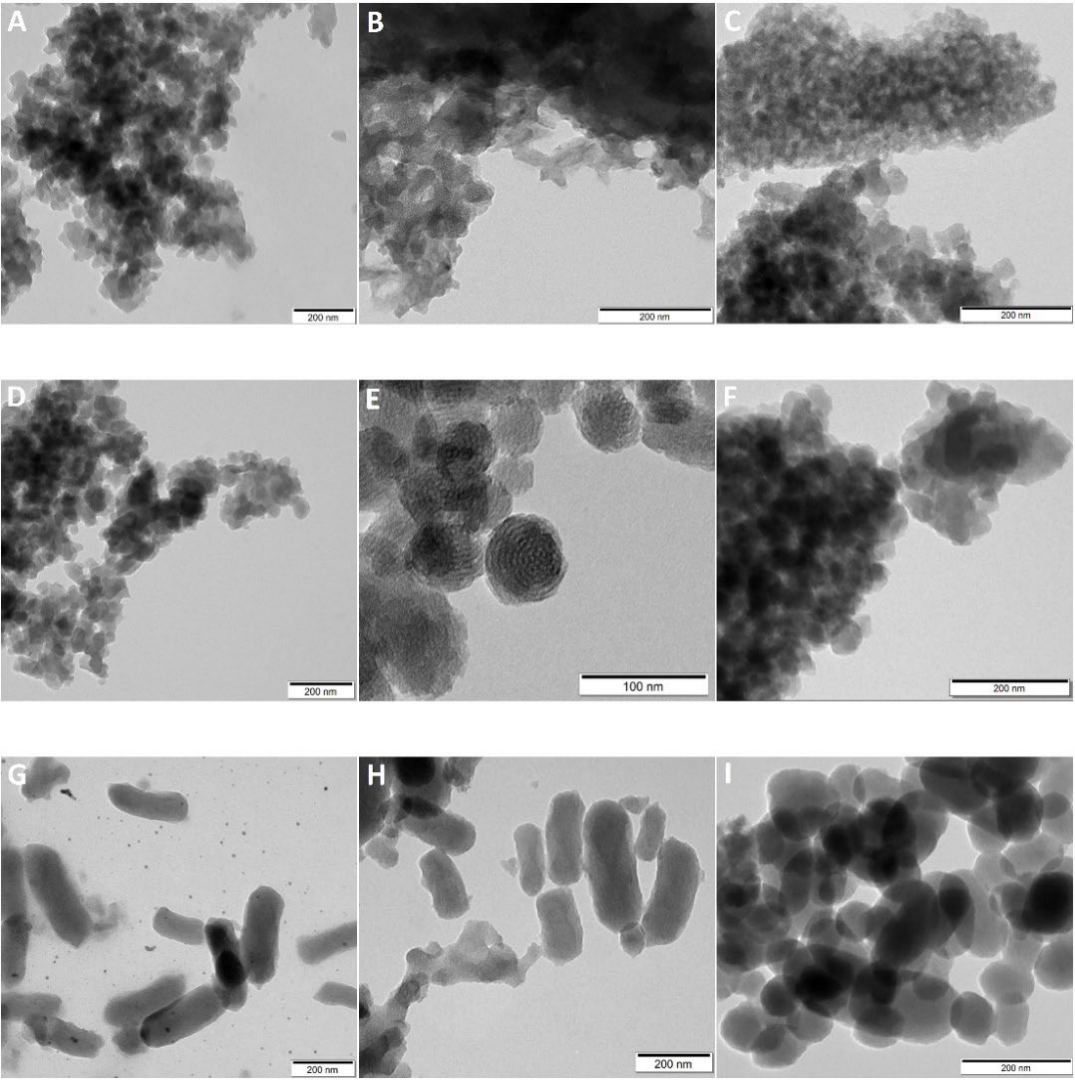
TEM images of particles: (**A**) S−SR, (**B**) S−NH_2_−SR, (**C**) S−Gal−SR, (**D**) M−SR, (**E**) M−NH_2_−SR, (**F**) M−Gal−SR, (**G**) L−SR, (**H**) L−NH_2_−SR, (**I**) L−Gal−SR.

**Figure 3:**
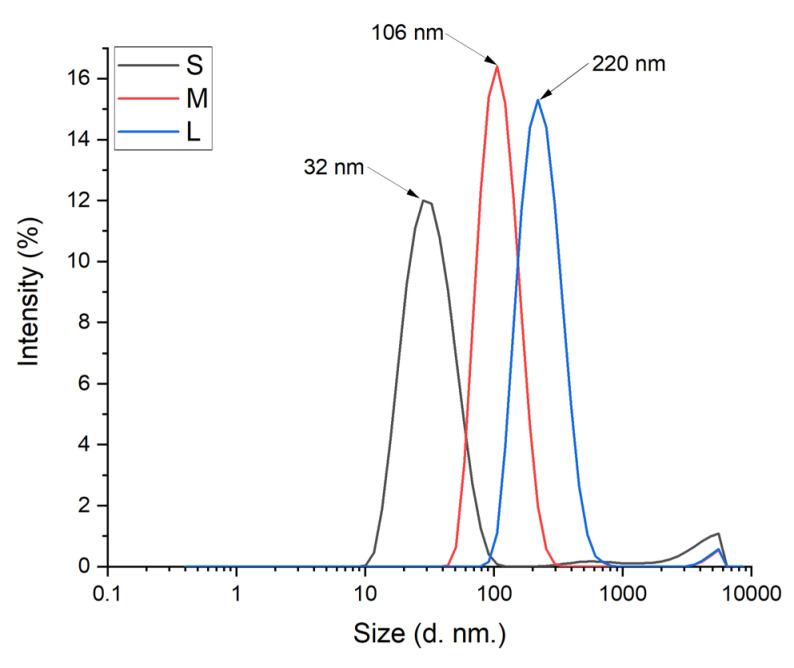
Particle size distribution obtained from DLS measurements.

The particle size was confirmed directly by TEM (Figure 2) imaging and the results were supported indirectly by dynamic light scattering (DLS) analysis (Figure 3). The obtained particles were 32 nm (PDI = 0.261) (for the ‘S’ materials), 106 nm (PDI = 0.163) (for the ‘M’ materials) and 220 nm (PDI = 0.165) (for the ‘L’ materials) in diameter, respectively. The particle sizes observed in the TEM images were slightly smaller than the particle sizes determined by the DLS studies due to the differences in the analytical principle used in both methods. The size of the particles obtained from DLS analysis is the hydrodynamic diameter, which takes into account the diameter of the particle along with the solvation layer adsorbed on its surface.

**Figure 4:**
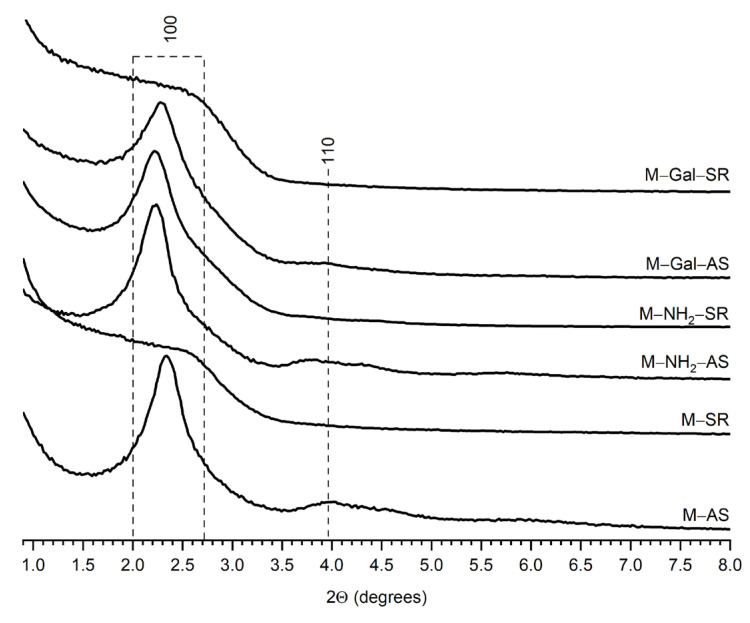
Low angle PXRD patterns of the ‘M’ particles functionalised with APTES (M−NH_2_) and galactose (M−Gal) before (AS) and after (SR) template removal.

Low angle PXRD (Figure 4, Supplemcentary Materials, Figures S2 and S4) measurements were also performed to confirm the hexagonal arrangement of the pores before and after surfactant removal. Three intense peaks indexed as reflections (100), (110) and (200) characteristic of MCM−41 type materials were observed, confirming the hexagonal arrangement of the pores. The pore architecture was preserved as evidenced by the contrast noted in the structure in the TEM images (Figure 2). The ‘L’ particles, despite the altered morphology to a rod-like shape, did not differ from the other particles in pore architecture.

**Figure 5:**
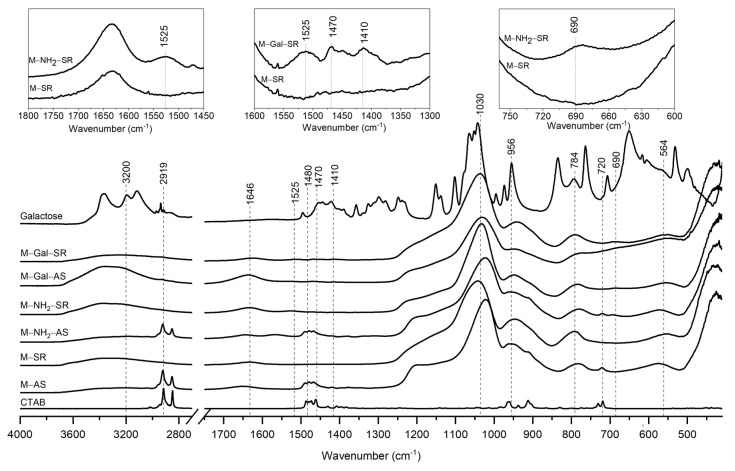
FTIR spectra of the ‘M’ particles functionalised with APTES (M−NH_2_) and galactose (M−Gal) before (AS) and after (SR) template removal. FTIR spectra of CTAB and galactose are given for comparison. The insets at the top of the figure represent the zoomed spectra with peaks that prove the presence of the NH_2_ and Gal functionalities.

The strong FTIR vibrational bands of the synthesised materials (M−SR, and M−AS, Figure 5, for ‘S’ and ‘L’ materials see Supplementary Materials Figures S1 and S3) characteristic of asymmetric stretching vibrations of Si-O-Si groups were centred at 1030 cm^−1^ and 784 cm^−1^. The peaks at 564 cm^−1^ and 426 cm^−1^ corresponded to the bending vibrations of Si−-O−Si groups. The peak seen at 956 cm^−1^ was attributed to the stretching of the Si−OH bond. The observed broad peaks centred at 3200 cm^−1^ indicated O−H groups and adsorbed water. Similarly, the peak at 1646 cm^−1^ corresponded to the bending vibrations of the O−H group of adsorbed water.

In the spectra of AS materials, the peaks located at 2919 cm^−1^, 1480 cm^−1^ and 720 cm^−1^ corresponded to the surfactant template (CTAB) present in the silica pores. Their disappearance in the spectra of SR−labelled materials indicated the successful removal of the template.

### 2.2. Surface Functionalisation with GAL and LAC

MSN functionalised with galactose (‘S’, ‘M’ and ‘L’) or lactose (only ‘M’ particles) displayed no change in particle morphology while increased aggregation was observed across all functionalised materials (Figure 2). Furthermore, TEM images confirmed the presence of the pores in the functionalised materials. This was corroborated with N_2_ adsorption–desorption isotherms (Supplementary Materials, Figure S5 and Table S1). The presence of the reflections (100), (110) and (200) in low angle PXRD (Figure 4, Supplementary Materials Figures S2 and S4) traces confirmed the presence of the hexagonal arrangement of the pores in the functionalised particles while some loss of signal intensity was observed after surfactant removal. Loss of signal intensity was associated with the difference in the scattering power/scattering contrast between the amorphous silicate wall and amorphous sorbate in the mesoporous structure as well as the presence of organic groups close to the pore openings.

To confirm the presence of NH_2_, Gal and Lac groups at the surface of functionalised materials, FTIR and NMR analyses were performed. Although FTIR is frequently used to confirm the presence of organic groups at the silica surface, for the materials that have an external surface that is exclusively functionalised the application of this method is challenging due to (i) the low content of organic functionalities, i.e., <3%, of the material mass as well as (ii) substantial broadening of the peaks that represents a lack of long-range ordering. The low intensity bands at wavenumbers of 1525 cm^−1^ and 690 cm^−1^ indicated the successful attachment of the amino groups (Figure 5 insets, Supplementary Materials Figures S1 and S3). The appearance of the vibrational bands at 1470 and 1410 cm^−1^ can be assigned to the presence of galactose molecules at the silica surface (Figure 5 insets, Supplementary Materials Figures S1 and S3). It is important to notice that the reductive amination of galactose led to the opening of the galactose ring (Figure 1). Therefore, it is expected that some of the FTIR peaks characteristic for galactose will disappear or undergo substantial shifts, making the molecular level characterisation of the functionalised materials based on FTIR spectra extremely challenging.

**Figure 6:**
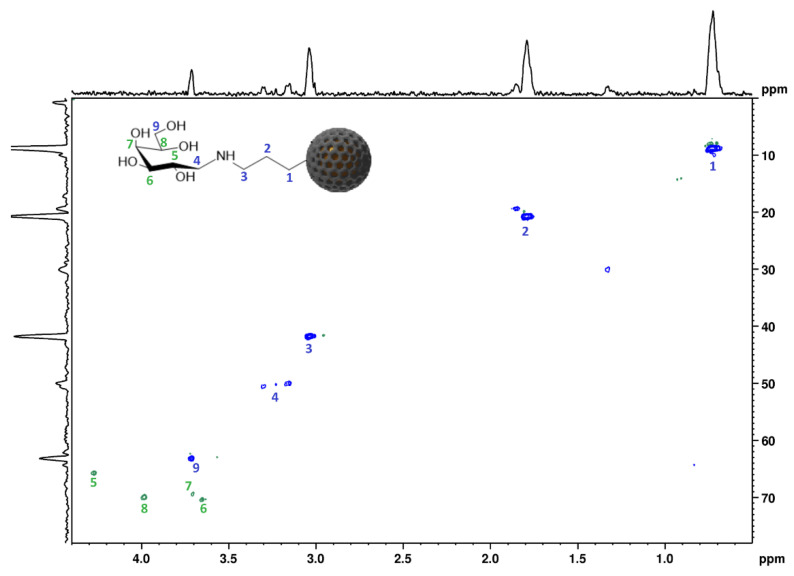
^1^H-^13^C HSQC HR-MAS NMR spectrum of galactose functionalised M−GAL−SR particles. The spectra assignment was made based on the NMR spectra predictor available at nmrdb.org (accessed on 20 March 2022) as well as using the reference spectra available at hmdb.ca (accessed on 20 March 2022) [33,34,35].

In contrast, the solid-state ^1^H-^29^Si CP/MAS and ^1^H-^13^C HSQC NMR spectrum of M−Gal−SR particles obtained under HR-MAS conditions provided a wealth of structural information. At least nine distinct signals were observed in the spectrum (Figure 6, see Supplementary Materials Figures S6–S9 for ‘S’ and ‘L’ particles) proving the successful functionalisation with the aminopropylene group (peaks 1, 2 and 3), followed by the attachment of the galactose functionality (peaks 4–9) via reductive amination. In comparison to the neat galactose ^1^H-^13^C HSQC spectrum (Supplementary Materials, Figure S7), in the spectrum of the M−Gal−SR particles, a substantial shift of anomeric carbon towards a higher field (ca. 40 ppm shift in ^13^C spectrum; ca. 2 ppm in ^1^H spectrum) was observed (peak 4), indicating an opening of the galactopyranose ring following the attachment of amine. Furthermore, peak 4 was composed of three resonances similarly to peak 2 in the aminopropylene group. This may indicate slight differences in the environment of both sites once being grafted as T1, T2 or T3 sites that have frequently been described for organofunctionalised mesoporous silica materials [25]. The ^1^H-^29^Si CP/MAS NMR spectrum displayed only Q sites for neat L−SR particles while the presence of T sites was observed in the spectra of L−NH_2_−SR and L−Gal−SR particles (Supplementary Materials, Figure S6).

**Figure 7:**
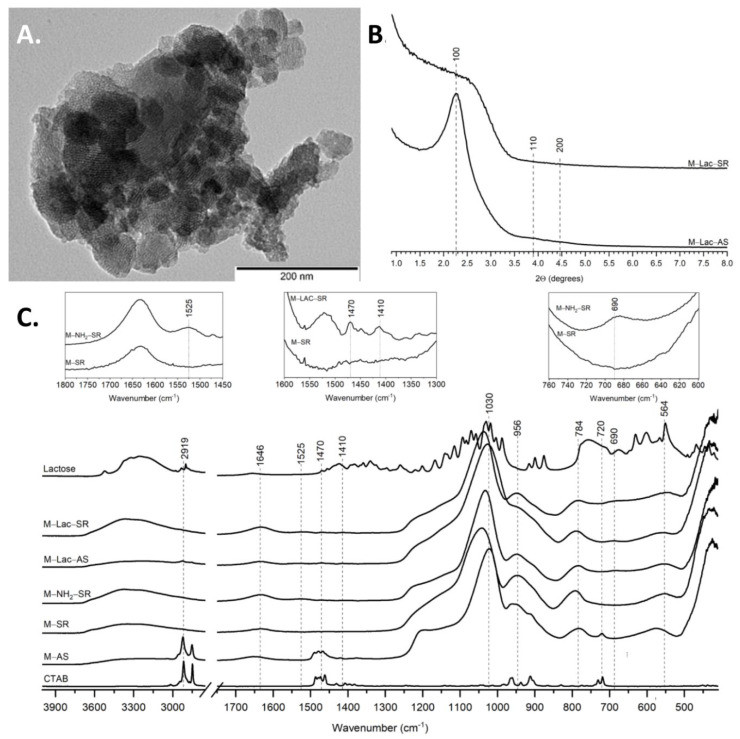
(**A**) TEM image of M−Lac−SR particles; (**B**) low angle PXRD patterns of M−Lac particles before (AS) and after (SR) template removal; (**C**) FTIR spectra of ‘M’ particles functionalised with APTES (M−NH_2_) and lactose (M−Lac) before (AS) and after (SR) template removal. FTIR spectra of CTAB and lactose are given for comparison. The insets at the top of the figure represent zoomed spectra with peaks that prove the presence of NH_2_ and lactose functionalities.

In order to investigate the possibility of attaching galactose to the material while preserving its structure (without breaking the ring), the functionalisation of ‘M−NH_2_−AS’ particles with lactose (a sugar composed of galactose and glucose units), was performed (Figure 7). This system also enabled us to evaluate the applicability of HR-MAS NMR spectroscopy for a more complex system that has disaccharide at the surface (at least 15 sites were expected in the spectrum). The TEM image (Figure 7A) confirmed the preservation of particle structure and morphology and the presence of the pores after functionalisation and surfactant removal. The FTIR analysis (Figure 7C) confirmed the presence of amino functionality (peaks at 1525 and 690 cm^−1^) as well as the presence of lactose (low intensity, broad bands at 1470 and 1410 cm^−1^). The PXRD patterns (Figure 7B) of the particles after surfactant removal displayed a decrease and a broadening of the peaks that could be attributed to the loss of signal intensity due to the difference in the scattering power/scattering contrast between the amorphous silicate wall and the amorphous sorbate in the mesoporous structure.

**Figure 8:**
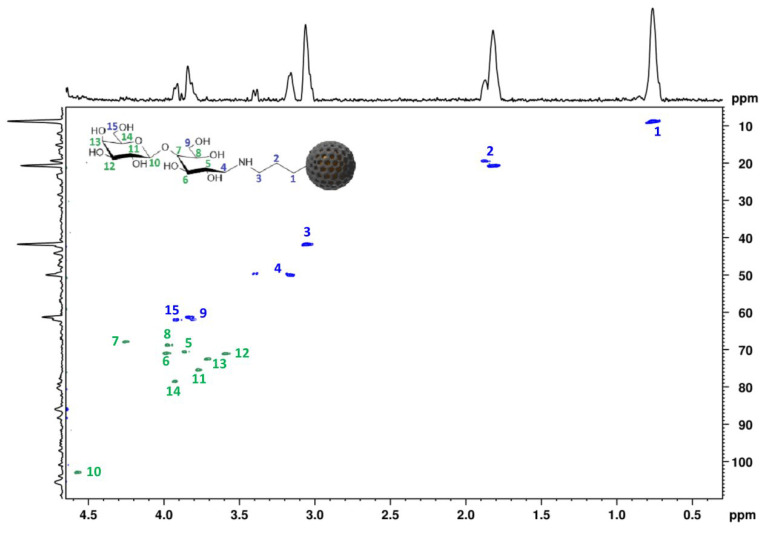
^1^H-^13^C HSQC HR-MAS NMR spectrum of M−Lac−SR particles. The spectra assignment was made based on the NMR spectra predictor available at nmrdb.org (on 20 March 2022) as well as the use of the reference spectra available at hmdb.ca (on 20 March 2022) [33,34,35].

The functionalisation of the particle surface with lactose was also confirmed by a HSQC NMR study (Figure 8). The obtained spectrum displayed 15 signals that were assigned to the structure of lactose-functionalised particles. In comparison to the spectra of neat lactose (Supplementary Materials, Figure S10), the substantial shift of anomeric carbon of glucose towards a higher field (ca. 40 ppm shift in ^13^C spectrum; ca. 2 ppm in ^1^H spectrum) was observed (peak 4) indicating an opening of the glucose ring following the attachment of amine, leaving the galactose ring intact. This may be of great importance in the development of novel galactose-functionalised MSN-based carriers for targeted delivery to the liver [21,36,37]. Similar to the galactose-functionalised particles, some of the peaks were present in more than one environment which can be attributed to the different binding motifs of APTES to silanol groups.

## 3. Materials and Methods

Three types of MCM−41 silica nanoparticles differing in size (S—32 nm, M—106 nm, L—220 nm) were synthesised using the sol-gel method. All chemicals were purchased from Sigma-Aldrich, USA. Tetraethoxysilane (TEOS) was used as the silica precursor and hexadecyltrimethylammonium bromide (CTAB) was used as the template, while (3-aminopropyl)triethoxysilane (APTES), D-(+)-galactose and lactose were used to functionalise the material surface in the presence of sodium cyanoborohydride (NaBH_3_CN) and a borate buffer.

### 3.1. MSN Synthesis

MCM−41 type MSN were synthesised (see Scheme 1) according to the previously reported procedure [38]. The NH_4_OH solution was prepared by diluting 25% ammonia solution in water. The pH was adjusted to 11.00, 11.32 and 11.38 in order to provide the control over materials size (particles labelled S—small, M—medium, L—large). The solution was heated to 50 (S−particles), 40 (M−particles) or 30 (L−particles) °C and CTAB was added and stirred until dissolved. In a separate beaker, 0.88 M ethanolic TEOS solution was prepared and added dropwise into ammonia water. The solution was stirred for 1 h. After this time stirring was turned off and the solution was allowed to age at a constant temperature. After 18 h, the product (AS—as synthesised, S−AS, M−AS, L−AS) was filtered, washed with deionised water and ethanol and dried. The obtained material was divided into two parts. One was destined for subsequent functionalisation and the remaining part was extracted for 24 h in a mixture of ethanol and hydrochloric acid to remove the surfactant. The product (SR—surfactant removed, S−SR, M−SR, L−SR) was filtered, washed with water and ethanol and dried.

### 3.2. MSN Functionalisation

To functionalise the external surface of the nanoparticles, a grafting method was used. Parts of dried, templated particles of a given size (S−AS, M−AS, L−AS) were suspended in 100 mL of toluene. Then (3-aminopropyl)triethoxysilane (APTES) was added and allowed to stand for 20 h. After this time, the product (labelled: S−NH_2_−AS, M−NH_2_−AS, L−NH_2_−AS) was filtered, washed several times with ethanol and deionised water and allowed to dry. One part of the dried material was destined for further functionalisation, and the other was subjected to template removal in a mixture of ethanol and HCl following the procedure described above in the Section 3.1. MSN Synthesis.

Gal/Lac were grafted on the silica surface by reductive amination using NaBH_3_CN as a reducing agent. The obtained particles (S−NH_2_−AS, M−NH_2_−AS, L−NH_2_−AS) were suspended in a borate buffer (pH 9). Then 50 mg of galactose was added and stirred for 15 min. After this time, sodium cyanoborohydride (NaBH_3_CN) was added to the mixture and stirred overnight. The product (S−Gal−AS, M−Gal−AS, L−Gal−AS) was filtered, washed several times with ethanol and water and dried. The surfactant was removed by 24 h extraction in a mixture of ethanol and HCl. The final product (S−Gal−SR, M−Gal−SR, L−Gal−SR) was filtered, washed extensively with water and ethanol and dried. For lactose functionalisation, M−NH_2_−AS particles were selected. All steps were performed in the same manner as for galactose functionalisation, the amount of added lactose was 95 mg and the resultant products were labelled M−Lac−AS and M−Lac−SR.

### 3.3. Particle Size and Shape

Hydrodynamic diameters of the synthesised particles were determined by dynamic light scattering. The measurements were performed with Zetasizer Nano ZS ZEN3600 (Malvern Instruments Ltd., Malvern, UK) equipped with a laser light source (λ = 633 nm) and a detector operating at a 173° scattering angle. The 1 mL samples were taken directly from the synthetic mixture at the end of the aging step and investigated immediately. Experiments were run at 25 ± 0.1 °C. Each sample was measured at least 5 times. The values of hydrodynamic diameters presented in this work are based on the intensity weighted size distribution.

The materials after filtration, functionalisation and surfactant removal were imaged with transmission electron microscopy (TEM; Hitachi H-800 microscope) providing a direct measure of the diameter of the particles as well as the presence of pores within the silica. The materials prior to the analysis were dispersed in ethanol and a drop of the obtained suspension was placed on the carbon on copper grids (Agar S160) and analysed after solvent evaporation.

### 3.4. Low Angle Powder X-ray Diffraction

The architecture of the hexagonal pores was confirmed using low angle PXRD (D2 Phaser Bruker, Billerica, MA, USA) with Cu Kα radiation (1.5418 Å) and a LynxEye detector. Each material was analysed in the range of 2θ 0.65–8° with 0.02° increment and 1 s irradiation time per step.

### 3.5. N_2_ Adsorption

Nitrogen adsorption−desorption isotherms for S−SR, M−SR, L−SR, L−NH_2_−SR and L−Gal−SR particles were measured using an autosorb iQ gas sorption system and ASiQwin software (Quantachrome Instruments, Boynton Beach, FL, USA) or ASAP 2020 (Micromeritics, Norcross, GA, USA) at 77 K. Samples were outgassed under a high vacuum at 313 K for 16 h prior to the analysis. The BET specific surface area (S_BET_) and the volume of monolayer coverage were determined using the Brunauer–Emmett–Teller (BET) equation [39] in the linear range of the adsorption curve (0.1–0.25 P/P_0_). The pore size and distribution curves were calculated using N_2_ adsorption on silica at 77 K and the NLDFT cylindrical pore equilibrium model as implemented in the ASiQwin™ software (ver. 5.2, Quantachrome Instruments, Boynton Beach, FL, USA).

### 3.6. Fourier Transformed Infrared Spectroscopy (FTIR)

FTIR spectra of the as−synthesised (AS), surfactant−removed (SR) and functionalised materials were acquired using a Nicolet iS50 FTIR spectrometer (Thermo Scientific, Waltham, MA, USA) in the attenuated total reflectance (ATR) mode. All measurements were carried out in the wavelength range of 400–4000 cm^−^^1^ at a 4 cm^−^^1^ resolution with at least 512 scans per measurement.

### 3.7. Nuclear Magnetic Resonance (NMR) Spectroscopy

All HR-MAS and solution-state NMR spectra were acquired using a Bruker AVANCE III™ NMR spectrometer, consisting of the 54 mm bore superconducting, actively shielded Ascend™ magnet operating at 600.13 MHz and 150.91 MHz for ^1^H and ^13^C, respectively. The spectra of the functionalised materials were acquired using a dual inverse high-resolution magic angle spinning (HR-MAS) probe ^1^H/^13^C, ^2^H-lock; (optimised for ^1^H observation); equipped with a single axis magic angle gradient. The samples were packed in Kel-F inserts, soaked with D_2_O until wetted and transferred to 4 mm zirconia rotors. The 1D ^1^H MAS and 2D ^1^H-^13^C HSQC spectra of the materials were acquired at 4 kHz MAS rate.

^29^Si solid-state NMR spectra of L−SR, L−NH_2_−SR and L−Gal−SR particles were acquired using a Bruker 300 MHz Avance III spectrometer with a double resonance probe operating at frequencies 300.13 MHz and 59.63 MHz for ^1^H and ^29^Si, respectively. The materials were packed into Kel-F inserts and transferred to the 4 mm zirconia rotors and analysed at an MAS rate of 4 kHz. The materials were characterised using ^1^H-^29^Si (CP/MAS) NMR (^29^Si π/2 pulse length 4.5 μs with a contact time of 2 ms, SPINAL64 decoupling was used during signal acquisition, 2048 transients were acquired with a recycle delay of 30 s). The Hartmann–Hahn conditions for ^1^H-^29^Si CP/MAS NMR experiments were set with kaolinite. The ^29^Si chemical shifts were recorded with respect to TMS.

## 4. Conclusions

Three types of MCM−41 type mesoporous silica nanoparticles, differing in size (S—32 nm, M—106 nm, L—220 nm), were synthesised and their surface was functionalised with galactose (‘S’, ‘M’ and ‘L’ particles) or lactose (‘M’ particles). The synthesised materials displayed a narrow particle size distribution (‘S’ particles PDI = 0.261; ‘M’ particles PDI = 0.163; ‘L’ particles PDI = 0.165) and a hexagonal arrangement of pores with a diameter of ca. 3 nm. The pore architecture and the structure and morphology of the particles did not change after surface functionalisation with functional groups. The presence of saccharides covalently attached at the materials surface was confirmed using the combined application of FTIR and HR-MAS NMR spectroscopy. The use of HR-MAS NMR spectroscopy for the characterisation of organic groups attached at the silica surface enabled the successful functionalisation of the particles to be confirmed. High resolution 2D ^1^H-^13^C HSQC spectra were obtained within 2 h compared to the few days that would be required to acquire ^1^H-^13^C HETCOR solid-state NMR spectrum. Wider application of HR-MAS NMR may enable the development of new spectroscopic methods to study the interactions of targeted nanocarriers with proteins and complex biological systems in biorelevant media.

## Data Availability

The data supporting the reported results are available upon request from the authors.

## Supplementary Materials

The following supporting information can be downloaded at: https://www.mdpi.com/article/10.3390/ijms23115906/s1.
