# Supplementary material for: Molecular Level Characterisation of the Surface of Carbohydrate-Functionalised Mesoporous silica Nanoparticles (MSN) as a Potential Targeted Drug Delivery System via High Resolution Magic Angle Spinning (HR-MAS) NMR Spectroscopy
Source: Int J Mol Sci. 2022 May 25;23(11):5906. doi: 10.3390/ijms23115906 (PMC9180545; doi:10.3390/ijms23115906)
Supplement: Supplementary file 1 [file ijms-23-05906-s001.zip › ijms-1719133-supplementary.pdf]

Electronic supplementary information

Molecular Level Characterisation of the Surface of Carbohydrate-Functionalised *Mesoporous silica* Nanoparticles (MSN) as a Potential Targeted Drug Delivery System via High Resolution Magic Angle Spinning (HR-MAS) NMR Spectroscopy

Karolina Krajewska [1], Anna M. Gołkowska [1], Maciej Nowak [1], Marta Kozakiewicz-Latała [1], Wojciech Pudło [2], Andrzej Żak [3], Bożena Karolewicz [1], Yaroslav Z. Khimyak [4] and Karol P. Nartowski [1,*]

1 Department of Drug Form Technology, Wroclaw Medical University, Borowska 211A, 50-556 Wroclaw, Poland; karolina.krajewska@student.umw.edu.pl (K.K.); anna.golkowska@student.umw.edu.pl (A.M.G.); maciej.nowak@umed.wroc.pl (M.N.); marta.kozakiewicz-latala@umw.edu.pl (M.K.-L.); bozena.karolewicz@umed.wroc.pl (B.K.)

2 Department of Chemical Engineering and Process Design, Silesian University of Technology, M. Strzody 7 str., 44-100 Gliwice, Poland; wojciech.pudlo@polsl.pl

3 Faculty of Mechanical Engineering, Wroclaw University of Science and Technology (WUST), Wybrzeze Wyspianskiego 27, 50-370 Wroclaw, Poland; andrzej.zak@pwr.edu.pl

4 School of Pharmacy, University of East Anglia, Norwich Research Park, Norwich NR4 7TJ, UK; y.khimyak@uea.ac.uk

* Correspondence: karol.nartowski@umw.edu.pl; Tel.: +(48)-71-784-05-69

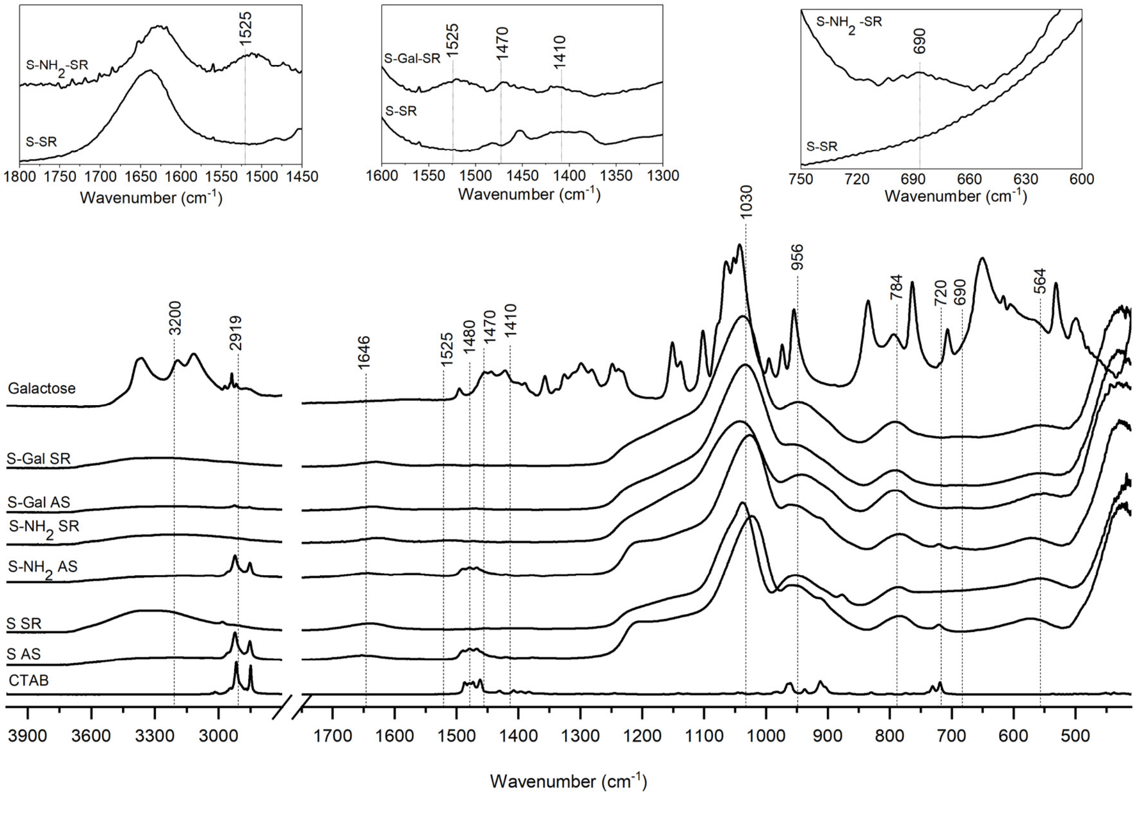

Figure S1. FTIR spectra of the 'S' particles functinalised with APTES (S-NH_2) and galactose (S-Gal) before (AS) and after (SR) template removal. FTIR spectra of CTAB and galactose are given for

comparison. The insets at the top of the figure represents zoomed spectra with peaks that prove the presence of NH_2 and Gal functionalities.

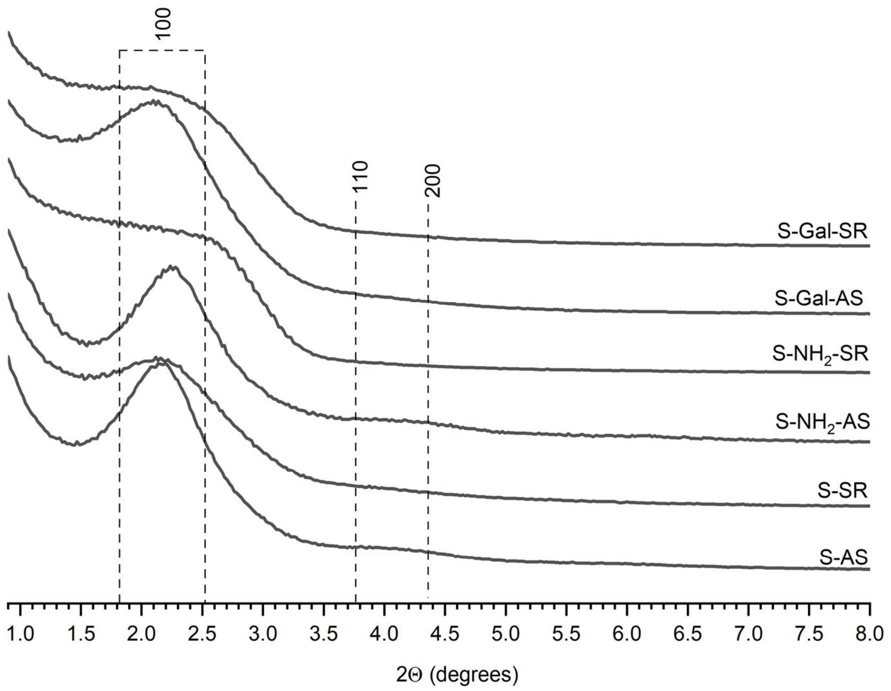

Figure S2. Low angle PXRD patterns of the ‘S’ particles functinalised with APTES (S-NH_2) and galactose (S-Gal) before (AS) and after (SR) template removal.

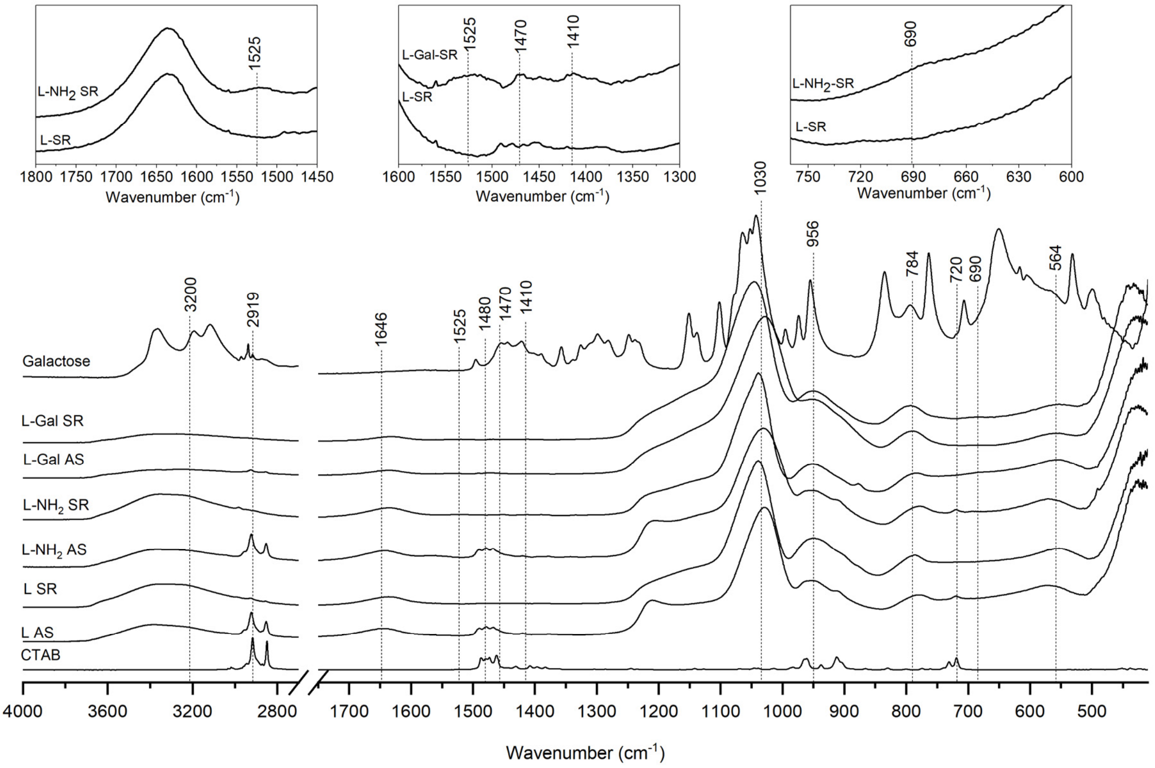

Figure S3. FTIR spectra of the 'L' particles functinalised with APTES (L-NH_2) and galactose (L-Gal) before (AS) and after (SR) template removal. FTIR spectra of CTAB and galactose are given for comparison. The insets at the top of the figure represents zoomed spectra with peaks that prove the presence of NH_2 and Gal functionalities.

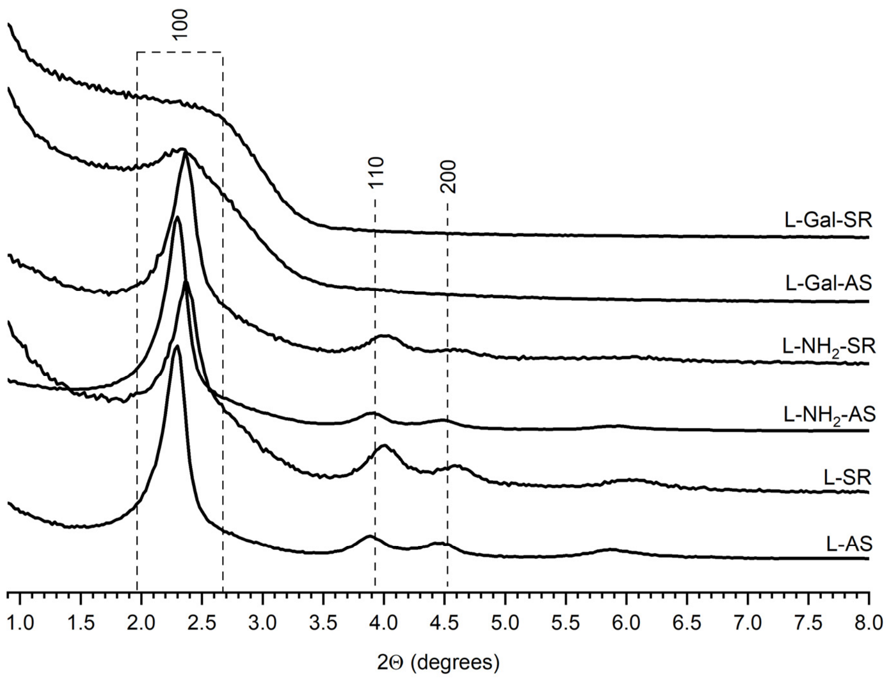

Figure S4. Low angle PXRD patterns of the 'L' particles functionalised with APTES (L-NH_2) and galactose (L-Gal) before (AS) and after (SR) template removal.

Table S1. Surface properties of the MSN materials

Material	BET surface area [m^2/g]	Average Pore Width [Å]	Pore Volume [cm^3/g]	Particle size[a] (nm)	PDI
S-SR	549	51	0.464	32	0.261
M-SR	204	32	0.447	106	0.163
L-SR	457	29	0.792	220	0.165
L-NH_2-SR	492	26	0.472	-	-
L-Gal-SR	442	31	0.551	-	-

[a] as determined for the as synthesised materials using DLS measurements

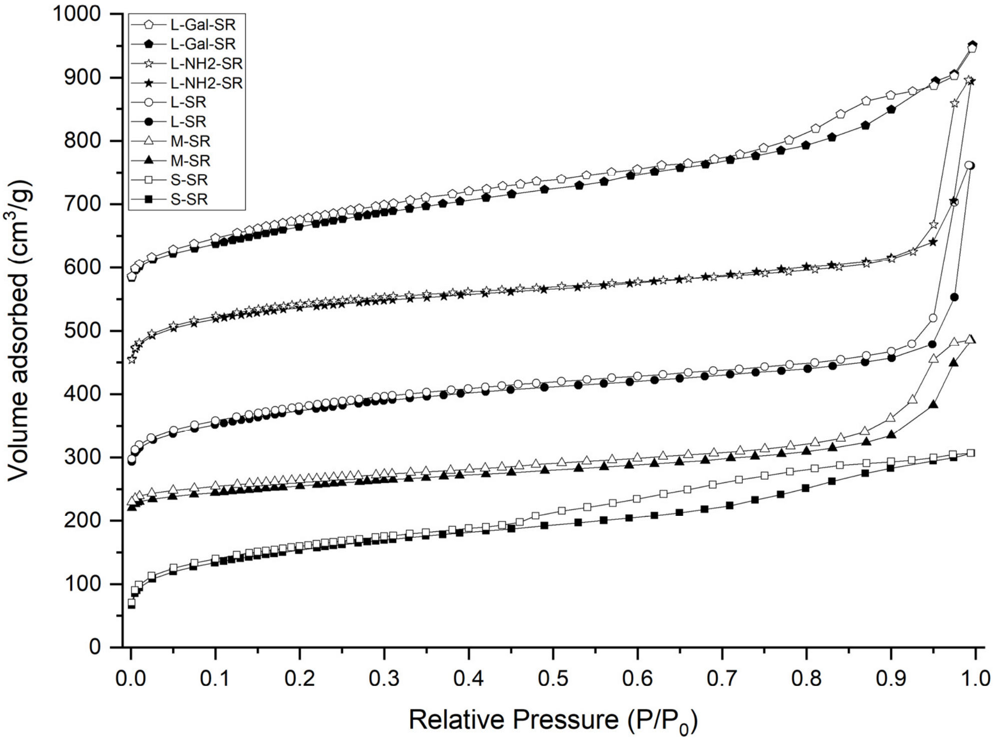

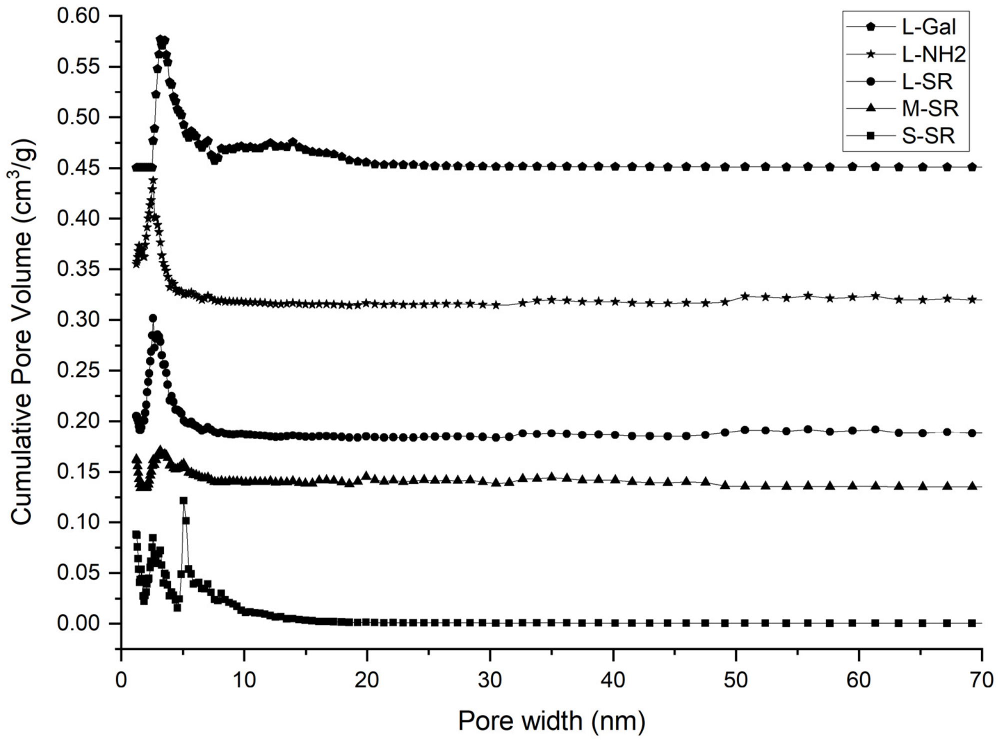

Figure S5. Nitrogen adsorption/desorption isotherms (top) and pore size distribution (bottom) of synthesized particles.

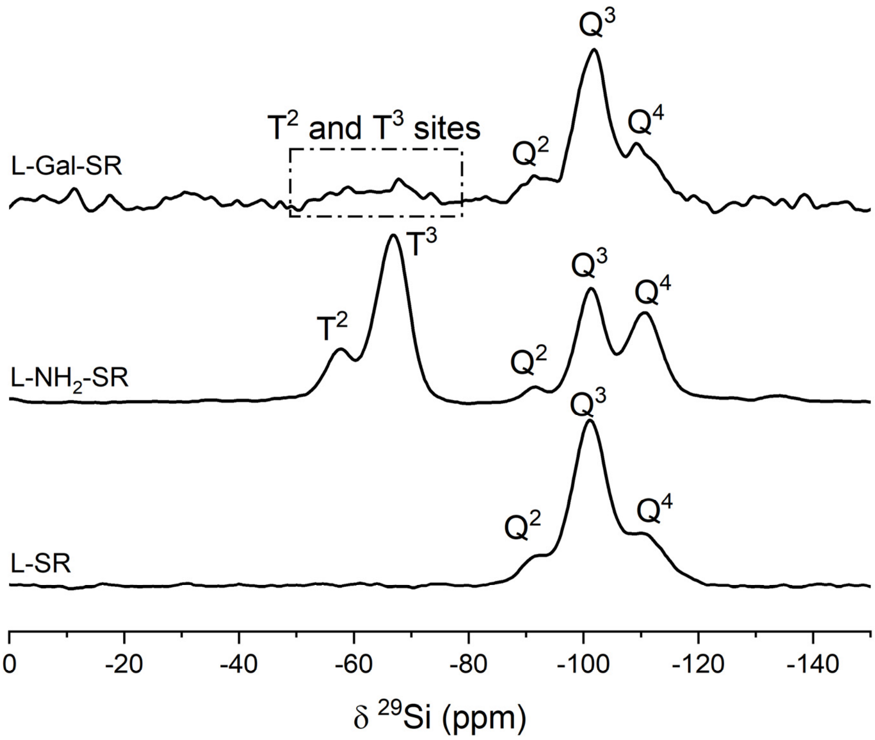

Figure S6. ^{1}H-^{29}Si CP/MAS NMR spectra of L-SR, L-NH_2-SR and L-Gal-SR particles.

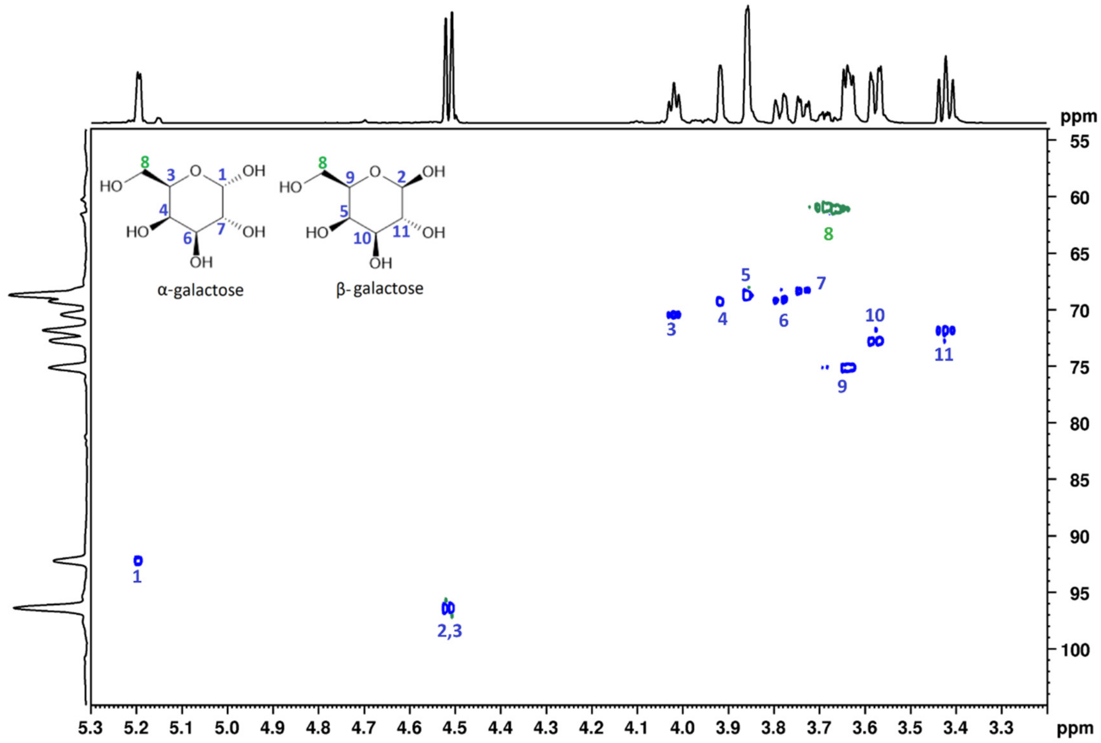

Figure S7. HSQC spectrum of galactose.

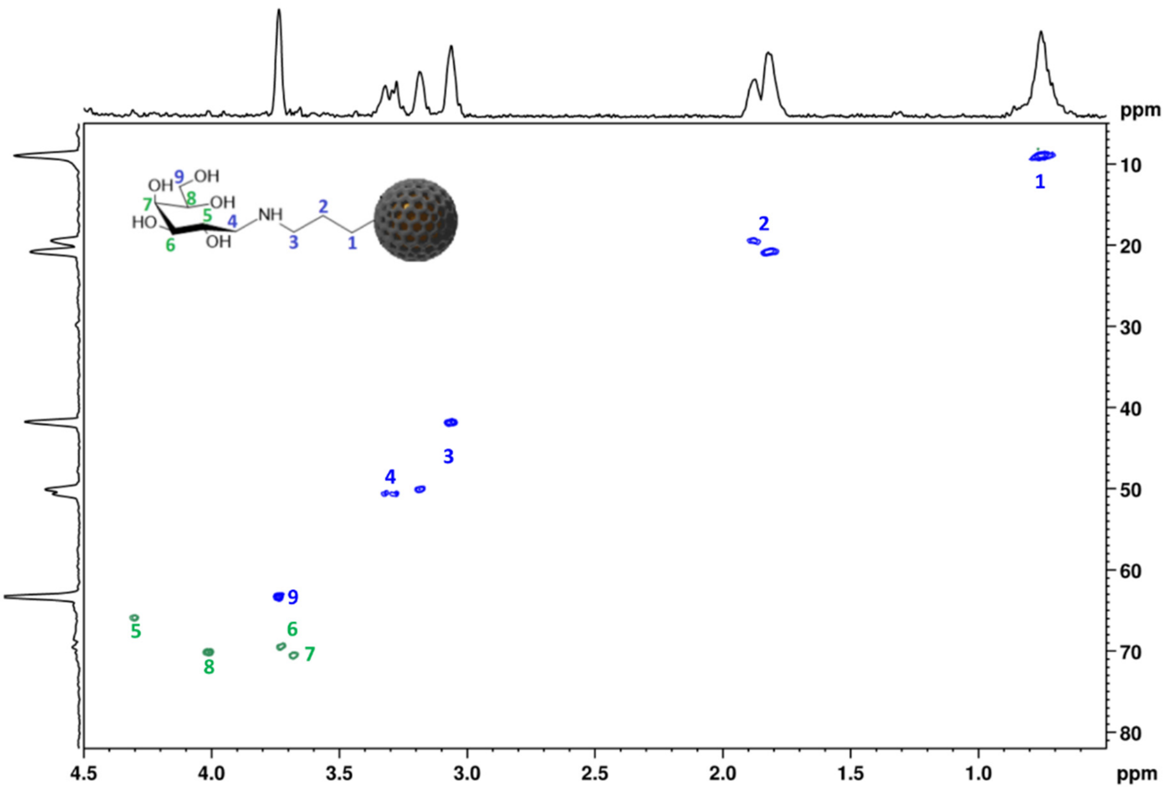

Figure S8. HSQC spectrum of galactose-functionalized "S" particles.

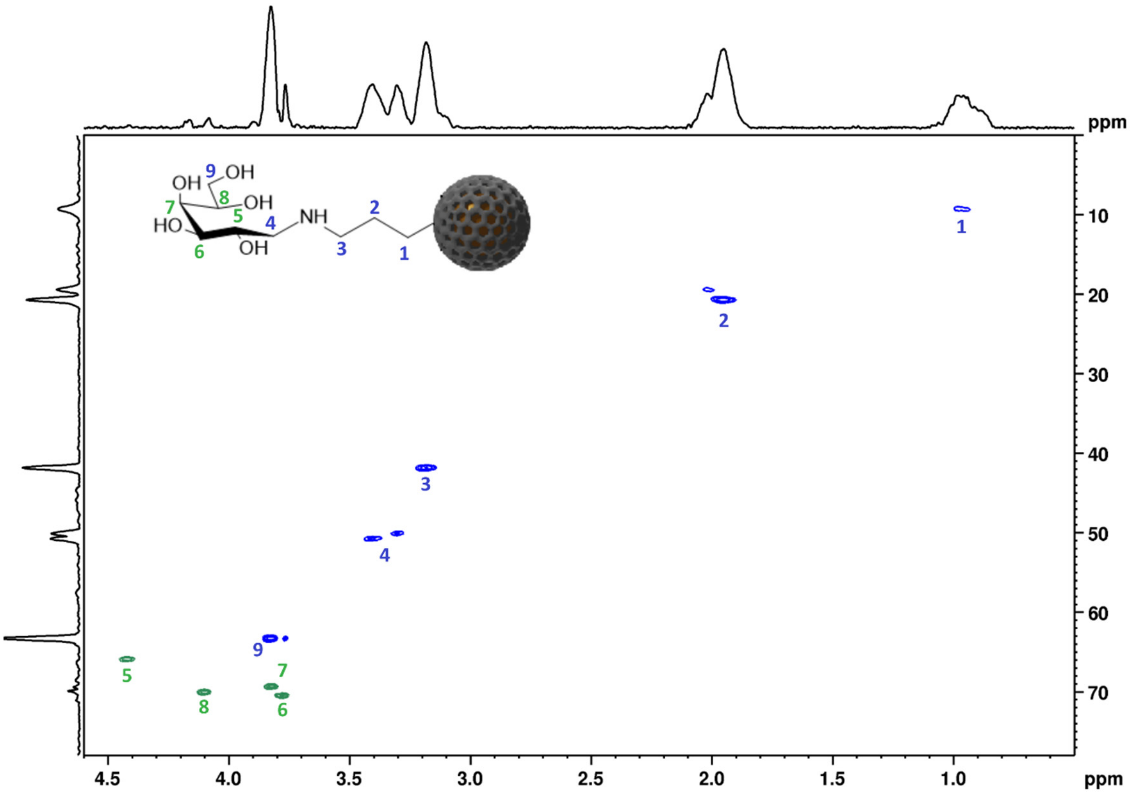

Figure S9. HSQC spectrum of galactose-functionalized "L" particles.

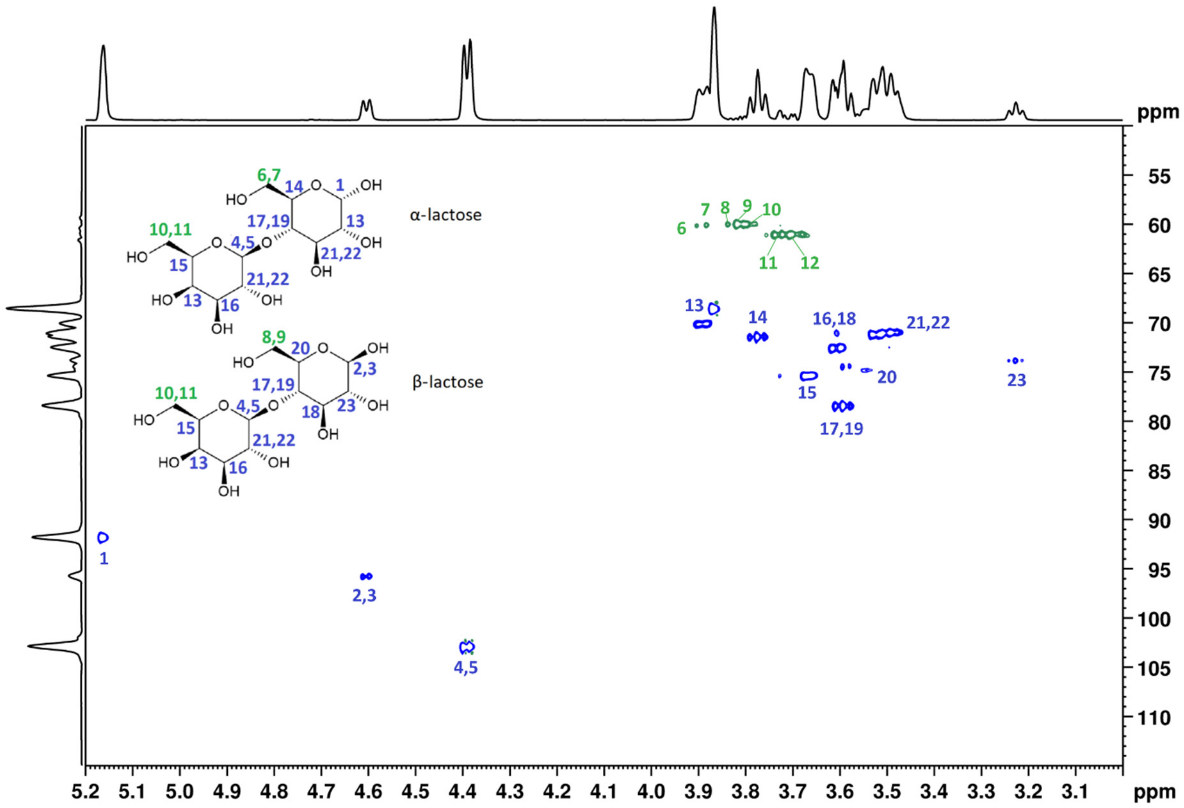

Figure S10. HSQC spectrum of lactose.
